# Masquelier’s grape seed extract: from basic flavonoid research to a well-characterized food supplement with health benefits

**DOI:** 10.1186/s12937-016-0218-1

**Published:** 2017-01-19

**Authors:** Antje R. Weseler, Aalt Bast

**Affiliations:** Department of Pharmacology and Toxicology; Faculty of Health, Medicine and Life Sciences, P.O. Box 616, 6200 MD Maastricht, The Netherlands

**Keywords:** Flavanols, Proanthocyanidins, Grape seed, Masquelier, Cardiovascular, Health, Inflammation, Oxidative stress, Nutraceutical, Food

## Abstract

Careful characterization and standardization of the composition of plant-derived food supplements is essential to establish a cause-effect relationship between the intake of that product and its health effect. In this review we follow a specific grape seed extract containing monomeric and oligomeric flavan-3-ols from its creation by Jack Masquelier in 1947 towards a botanical remedy and nutraceutical with proven health benefits. The preparation’s research history parallels the advancing insights in the fields of molecular biology, medicine, plant and nutritional sciences during the last 70 years. Analysis of the extract’s flavanol composition emerged from unspecific colorimetric assays to precise high performance liquid chromatography - mass spectrometry and proton nuclear magnetic resonance fingerprinting techniques. The early recognition of the preparation’s auspicious effects on the permeability of vascular capillaries directed research to unravel the underlying cellular and molecular mechanisms. Recent clinical data revealed a multitude of favorable alterations in the vasculature upon an 8 weeks supplementation which summed up in a health benefit of the extract in healthy humans. Changes in gene expression of inflammatory pathways in the volunteers’ leukocytes were suggested to be involved in this benefit. The historically grown scientific evidence for the preparation’s health effects paves the way to further elucidate its metabolic fate and molecular action in humans.

## Introduction

In scientifically evaluating the health benefit of a food-derived product, three major rules have to be followed: 1) The product for which a health claim is made should be well characterized; 2) the (claimed) effect should be well defined and of physiological benefit; 3) a cause-and-effect relationship between the intake of the food product and the claimed effect on human health is substantiated by human (observational and intervention) studies. In this review we will follow a specific botanical preparation, i.e. a mixture of monomeric and oligomeric flavan-3-ols from the seeds of grapes, on its travel from its creation in 1947 towards a botanical remedy and nutraceutical with proven health benefits.

The authentic mixture of monomeric and oligomeric flavan-3-ols extracted from the seeds of grapes (*Vitis vinifera* L.) is commercially available as the herbal remedy Endotélon® and as Masquelier’s® Original OPCs (Anthogenol®) in various food supplements. This plant extract forms an interesting example of how product-specific research followed and contributed to the scientific evolution of botanical, biochemical and physiological insights during the post-WWII period. In this time, grape seed extracts constantly remained at the forefront of successive innovations in scientific investigation [1]. Applications have been found in the treatment of chemo- and radiotherapy-induced toxicity [2], in chemoprevention [3–5], in cardiovascular diseases [6] and neurodegenerative diseases [7, 8], in oral health [9] or as cosmeceutical [10]. In the early development some focus was given to the effect of grape seed extracts as phlebotonics for venous insufficiency [11].

Research on the bioactive components of grape seeds extracts, i.e. in flavonoids, started in the beginning of the 20^th^ century, when nutritional scientists were dedicated to the isolation and identification of compounds we came to define as “vitamins.” The Hungarian scientist Albert Szent-Györgyi isolated vitamins C and P from citrus fruits. In contrast to vitamin C, the chemical characterization of vitamin P appeared to be difficult. As a result, it was not possible to attribute the vitamin P effect to a specific compound or preparation. Moreover, the notion of such a vitamin was abandoned since no deficiency disease could be linked to Szent-Györgyi’s citrus extract. However, the biological effect of “vitamin P”, i.e. its influence on vascular permeability was established as well as its enforcing influence on the anti-scurvy effect of vitamin C. Efforts to identify this putative vitamin P were directed to yellow colored plant pigments, the flavonoids (*flavus* (Latin) means yellow).

**Fig. 1:**
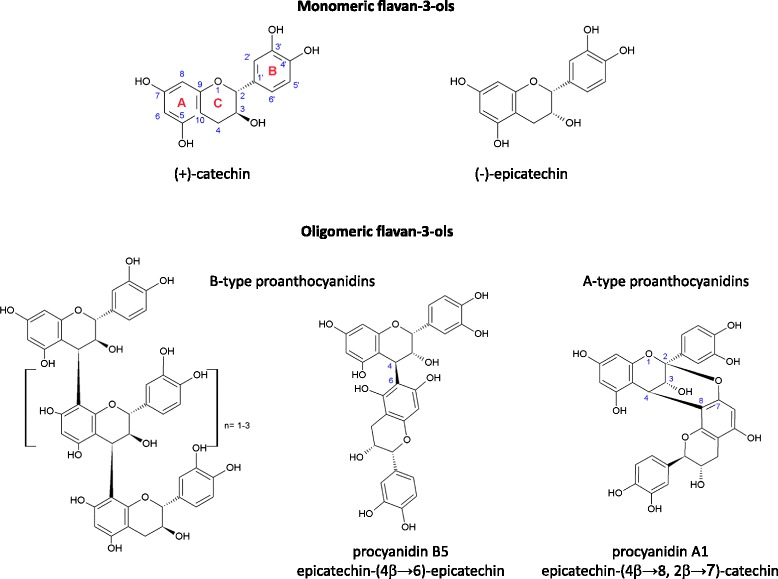
Molecular structure of monomeric and oligomeric flavan-3-ols

In 1947 the French researcher Jack Masquelier extracted during his work for his PhD thesis a colorless fraction from the red-brown skin of peanuts. He found this fraction to be responsible for the vitamin P effect in animals and proposed its major components as oligomers of flavan-3-ol units (i.e. (+)-catechin or (-)-epicatechin; Fig. 1). Nowadays, these polyphenolic compounds are commonly classified as monomeric and oligomeric flavan-3-ols. Condensed flavanols (oligomers and polymers) are also designated as proanthocyanidins. Oligomers of proanthocyanidins are well-known by the abbreviation “OPCs.” As the name suggests, proanthocyanidins are the precursors (*pro*) of anthocyani(di)ns (*anthos* (ancient Greek) means flower and *kyanos* (ancient Greek) means blue), which are water-soluble pigments being responsible for the blue, violet or red color of many flowers and fruits. In plants, anthocyanins are usually present as glycosides, i.e. bound to one or several sugar moieties. Their color depends on the pH of their environment changing from red/pinkish at acidic conditions to blue—purple at neutral conditions and yellowish at alkaline conditions.

In any case, the scientific journey in the field of extracts consisting of monomeric and oligomeric flavanols begins in the early attempt to classify these compounds as a vitamin amidst all other vitamins that were discovered in those days. As nutrients that are essential to prevent symptoms of deficiency, vitamins were realized to ensure and procure health. In this context, the first discovered health effects of Masquelier’s flavanol-preparations fitted well in the, at that time, new WHO definition of health as “a state of complete physical, mental and social well-being and not merely the absence of disease or infirmity” [12].

**Fig. 2:**
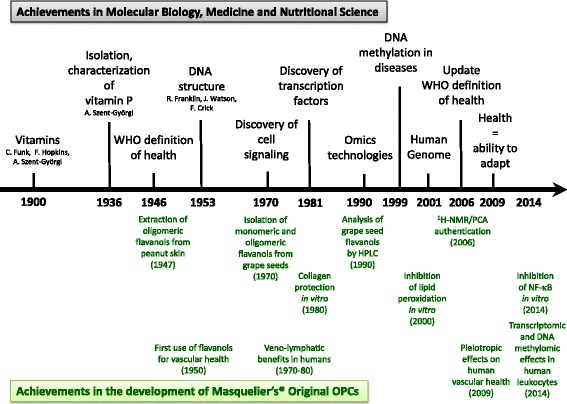
Timeline of achievements in the field of molecular biology, medicine and nutritional science during the last decade which are paralleled by achievements in the development and investigation of a grape seed extract by the French scientist Jack Masquelier. Today this preparation of monomeric and oligomeric flavan-3-ols is commercially available in the herbal remedy Endotélon® and as Masquelier’s® Original OPCs in nutraceuticals and food supplements

Besides developing synthetic routes [13] and improving methods for the extraction of monomeric and oligomeric flavanols from different plant sources, Jack Masquelier also directed much of his and his colleagues’ efforts to elucidate the biological activities of the extracts produced. In recent years, quite some scientific data have been generated that provide insights in the presumed effects of all sorts of more or less well defined grape seed extracts on human health. However, in contrast to drugs, which are mostly single chemical compounds, the evaluation of the biological effects of phytochemicals produced by extraction is challenging. The fact that most plant extracts contain a wide variety of individual chemical entities bedevils the establishment of a relationship between an effect in a biological test system and an individual, chemically-defined molecule which is part of the extract. For this reason, the extract as a whole has to be regarded as the biologically active entity. Therefore, to maintain and assure physiological consistency, it is essential to have a well-characterized and standardized plant extract available, of which each batch possesses the qualitative and quantitative composition of the researched preparation. Monomeric and oligomeric flavanols are heterogeneous phytochemicals that differ in their polymerization degree, stereochemistry and linkage of the monomeric units. As described later in detail, particular (parts of) plants are rich in these flavanols such as pine bark and grape seeds. A recently performed literature search in PubMed (http://www.ncbi.nlm.nih.gov/pubmed; search performed on 8 July 2016) using the search terms “grape” and “seed” and “extract” yielded 995 hits. However, a clear picture on the bioactivity and health effects of the category of extracts loosely defined as “grape seed-derived flavanols” can hardly be obtained, since data on the qualitative and quantitative composition of the investigated extracts are either missing, imprecise or incomparable. Therefore, in this review, we decided to focus on Masquelier’s grape seed-derived monomeric and oligomeric flavanols extract (Masquelier’s Original OPCs®), since it has a well-defined, consistent composition (*vide infra*) and an over decades established pool of in vitro, in vivo and clinical data unravelling health benefits of monomeric and oligomeric flavanols (Fig. 2).

*Many plant products originate from traditional use. Historical findings and concepts that come with it are a valuable source to get insides into the origin and potential health effects of these products. The data on Masquelier’s flavanol preparation suggest a vitamin-like action on capillary function, which can be associated with the monomeric and oligomeric flavanols present in this extract. A closer look at the biosynthetic origin of flavanols in plants may help to better understand their chemical nature and biological activity.*

### Flavanol biosynthesis in plants

**Fig. 3:**
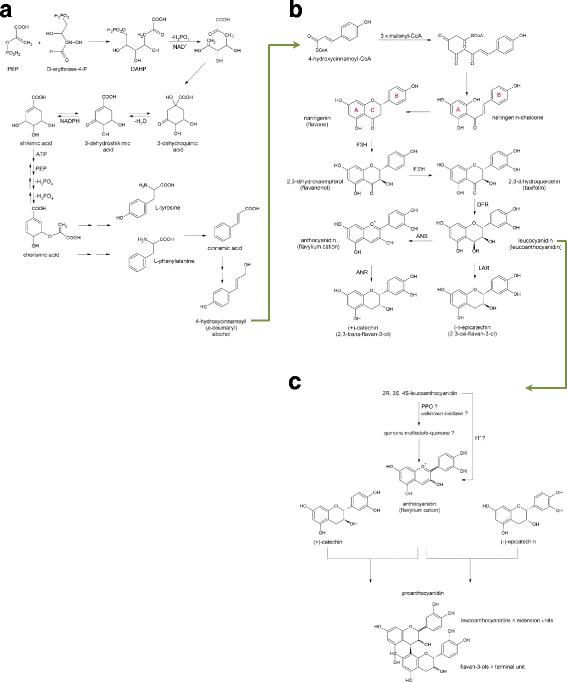
**a**-**c** Biosynthesis of monomeric and oligomeric flavan-3-ols in plants (panel **a**-**c**). **a** Shikimate pathway starting from phosphoenol pyruvate (PEP) and D-erythrose-4-phosphate (D-erythrose-4-P) leading to the formation of 4-hydroxycinnamoyl alcohol, DHAP = 3-deoxy-D-arabinose-heptulosonic acid-7-phosphate, NAD = nicotine-adenine dinucleotide, NADPH = reduced nicotine-adenine dinucleotide phosphate; **b** Formation of monomeric flavan-3-ols (+)-catechin and (-)-epicatechin from 4-hydroxycinnamoyl-CoA and 3 molecules of malonyl-CoA, F3H = flavanone-3-hydroxylase, F3’H = flavanone-3’-hydroxylase, DFR = dihydroflavanol-4-reductase, ANS = anthocyanidin synthase, ANR = anthocyanidin reductase, LAR = leucoanthocyanidin reductase; **c** Polymerization of monomeric flavan-3-ols to oligomeric proanthocyanidins, PPO = polyphenol oxidase, ? indicates uncertainty in existence/role of (intermediate) compound/enzyme

Monomeric flavan-3-ols like (-)-epicatechin and (+)-catechin are enzymatically synthesized by plants via a combination of the shikimate and the acetate pathway [14]. The first step towards the synthesis of shikimic acid consists of the condensation of phosphoenolpyruvate (PEP; produced in glycolysis) and D-erythrose-4-phosphate (produced in pentose phosphate cycle) to 3-deoxy-D-arabinose-heptulosonic acid-7-phosphate (DAHP; Fig. 3a). Intramolecular aldol-type condensation and NADH-mediated redox reactions result in dephosphorylation and cyclization to 3-dehydroquinic acid. Dehydration and reduction of the latter give shikimic acid which is eventually converted to chorismic acid. Chorismic acid serves as the precursor of the amino acids L-tyrosine and L-phenylalanine. From both amino acids 4-hydroxycinnamic acid (*p*-coumaric acid) is generated. Upon esterification with coenzyme A 4-hydroxycinnamoyl-CoA is extended by 3 malonyl-CoA units which are derived from the acetate pathway (Fig. 3b). This reaction is catalyzed by the enzyme chalcone synthase and leads via a polyketide intermediate to naringenin-chalcone. A Michael-type nucleophilic attack of one of the phenolic OH-groups on the α,β-unsaturated ketone results in the C-ring formation and yields the flavanone naringenin (Fig. 3b). Naringenin is the basic molecule from which all different flavonoid subclasses are synthesized including flavan-3-ols. Hydroxylation of naringenin by flavanone-3-hydroxylase (F3H) and subsequent NADPH-dependent reduction by dihydroflavanol-4-reductase (DFR) produces leucoanthocyanidins such as leucocyanidin. Leucoanthocyanidins can be converted by leucoanthocyanidin reductase (LAR) to 2,3-*trans*-(+)-flavan-3-ols like (+)-catechin. Moreover, they are oxidized by anthocyanidin synthase (ANS) to anthocyanidins which are reduced by anthocyanidin reductase (ANR) into 2,3-*cis*(-)-flavan-3-ols like (-)-epicatechin (Fig. 3b). The formation of 2,3-*cis* configured flavan-3-ols is remarkable because all chiral intermediates in the flavonoid biosynthesis pathway have a 2,3-*trans* configuration. Since the majority of proanthocyanidins are condensed products of (-)-epicatechin and (+)-catechin, it is obvious that the stereochemistry of the oligomers varies depending on the number and position of the (-)-epicatechin and (+)-catechin units. It is an ongoing debate if the polymerization of (-)-epicatechin and (+)-catechin units is facilitated by enzymes or occurs non-enzymatically [15]. Efforts to identify an enzyme that controls the condensation of the precursor units to proanthocyanidins have yet not been successful [16]. Polyphenol oxidase (PPO) has been suggested as a possible candidate (Fig. 3c) since it can catalyze the conversion of (-)-epicatechin and (+)-catechin to their corresponding quinone methides [17]. However, the physiological relevance of this mechanism remains doubtful because oligomeric flavanols are formed in the vacuole while PPO is usually located on plastids or chloroplasts [18]. An oxidase with similar catalytic activity in the vacuole has not been found until now.

Under acidic conditions in vitro proanthocyanidins could be synthesized from leucoanthocyanidins which condensate with (-)-epicatechin or (+)-catechin via the formation of a quinone methide and carbocation (flavylium cation) at the C4 position [19] (Fig. 3c). The electrophilic C4 of the leucoanthocyanidin (flavan-3,4-diol, i.e. the extension (upper) unit) reacts with the nucleophilic C6 or C8 position of the flavan-3-ol (i.e. the starter/terminal (lower) unit). However, the existence of an acidic compartment in plant cells was controversial until recent findings in *Arabidopsis thaliana* showed that gene disruption of the plasma membrane H^+^-ATPase AHA10 caused a substantial loss of proanthocyanidin accumulation in the seed coat endothelium [20]. Assuming that AHA10 or other plant H^+^-ATPases contribute to the acidification of cytoplasmic or vacuolar compartments, non-enzymatic condensation reactions in an acidic environment may occur in plants (Fig. 3c).

Despite many unresolved questions on the precise molecular processes leading to the proanthocyanidins formation in plants, a wealth of different structures has been identified so far. Based on their hydroxylation pattern oligomeric flavan-3-ols are classified in subgroups like procyanidins that have a 3’,4’-dihydroxylation pattern in the B-ring ((+)-catechin and (-)-epicatechin extension units), propelargonidins with 4’-hydroxylation pattern in the B-ring ((+)-afzelechin and (-)-epiafzelechin extension units) and prodelphinidins with 3’,4’,5’-trihydroxylation pattern ((+)-gallocatechin and (-)-epigallocatechin extension units). The hydroxylation pattern of the A and C-ring is identical in these 3 subgroups and comprises OH-groups at C3, C5 and C7. However, more proanthocyanidin-subgroups have been isolated from plants or chemically synthesized that differ in their positions of OH-groups in the A and C ring [16]. In accordance to the interflavan linkage procyanidins are classified in A- and B-type. Oligomers (and polymers) of the B-type are linked via 1 bond which is usually located between C4 of the extension (upper) unit and the C6 or C8 of the starting/terminating (lower) unit (e.g. procyanidin B5, Fig. 1). In addition to this C-C-linkage, A-type proanthocyanidins possess an ether bond between the C2 of the upper flavan-3-ol unit and the O at the C5 or C7 position of the lower unit (e.g. procyanidin A1, Fig. 1). In both types, the stereochemistry of the linking bond(s) between two and more flavan-3-ol units can be either α (denoted as hashed wedge in the molecular structural formula) or β (denoted as solid wedge). In order to cope with the increasing numbers of individual proanthocyanidins structurally elucidated in plants, a novel nomenclature has been introduced to unequivocally designate the complex molecular structures with higher polymerization degree [21]. The building units of the oligomers are named according to their names as monomeric flavan-3-ols framing parenthesis which specify the C-position and direction of the interflavan linkage indicated as an arrow. For example, the dimer procyanidin B1 is designated as epicatechin-(4β → 8)-catechin, the dimer procyanidin A1 (Fig. 1) is given as epicatechin-(4β → 8, 2β → 7)-catechin and the trimer procyanidin C1 is denoted as [epicatechin-(4β → 8)]_2_-epicatechin.

*Unravelling the biosynthesis of monomeric and oligomeric flavanols provides knowledge on the nature of the phytochemicals. In the case of monomeric and oligomeric flavanols it becomes obvious why they are not glycosylated in plants and why there exists a large variety of different oligomeric flavanols. These insights also clarify why it is an absolute necessity to carefully characterize commercially available flavanolic preparations used in medicines*, *food supplements and cosmetics. Batch*-*to*-*batch consistency allows establishing the cause*-*and*-*effect relationship between the product and its physiological effect, i.e. the physiological consistency of the preparation. Therefore*, *scientific data of a well*-*characterized flavanolic preparation cannot be extrapolated to or associated with the daily consumption of flavanols* via *ordinary foods*, *because foods contain a wide and unspecific variety of all kinds of flavanols. Dietary intake of flavanols is relevant only in the context of epidemiological studies in which average and mean intakes are associated with the health status of certain populations. In this respect the association between the consumption of red wine and cardiovascular mortality may serve as an example* [22]. *On top of that*, *during the past 30 years many more epidemiological data have become available on the dietary intake of flavanols and the health of populations*.

### World-wide daily flavan-3-ol and total flavonoid intake

Monomeric, oligomeric and polymeric flavanols form one of the most abundant classes of flavonoids and are almost ubiquitous in the plant kingdom. As such they are present in a wide variety of vegetables and plant-derived food products including wine, fruit juices, tea leafs, cocoa beans, fruits, cereal grains and legume seeds [23–25]. Among fruits, berries like blackcurrants, blueberries, chokeberries, strawberries have been found to contain the highest amounts of monomeric and oligomeric flavanols. Also apples and plums are sources of monomeric and oligomeric flavanols and are suggested to contribute to their dietary amount daily consumed [25]. By combining the latest food composition databases on polyphenols and using dietary recall data of large population cohort studies estimations of the habitual intake of monomeric and oligomeric flavanols in Europe were recently published [26–28]. Considerable differences in the dietary flavanol intake among European countries and geographic regions were observed. The average total flavan-3-ol intake in Europe was reported to be 369 mg/d [27] with the highest intake in Ireland (mean: 793 mg/d; median: 701 mg/d) and the lowest intake in the Czech Republic (mean: 181 mg/d; median: 69 mg/d). The mean and median intake of proanthocyanidins (both, oligomers (i.e. 2–10 flavanol units) and polymers (i.e. > 10 flavanol units)) in Mediterranean countries was calculated to 160 mg/d and 30 mg/d (highest intake in Spain; mean: 175 mg/d, median: 36 mg/d), respectively. This exceeded the mean and median intake of countries in central Europe (114 and 26 mg/d, respectively) and northern (Scandinavian) countries (110 and 25 mg/d, respectively). The lowest averaged proanthocyanidin intake of all European countries was found in The Netherlands with 96 mg/d (median: 19.6 mg/d). Tea, wine, cocoa products, pome (in particular apples and pears) and stone fruits as well as berries could be identified as major dietary sources of proanthocyanidins [26–28]. It was also observed that the proanthocyanidin intake within populations seems to depend on socio-demographic, anthropometric and lifestyle characteristics. Subjects with a higher level of education, increasing age (44–64 years), being moderately active and without obesity (i.e. body mass index (BMI) < 30 kg/m^2^) appeared to consume more proanthocyanidins than subjects without formal schooling, younger than 44 years, being inactive and having an BMI > 30 kg/m^2^ [28].

In contrast to Europe, adults in the USA seem to daily consume on average lower amounts of total flavonoids, flavan-3-ols and proanthocyanidins, respectively. Latest data from a 24 h food recall in a cohort of 5420 people above 20 years old indicated a mean total flavonoid intake of 251 mg/d of which 81% comprised flavanols mainly derived from tea [29]. The oligomeric proanthocyanidin intake was estimated to range between 60 and 95 mg/d [25, 30]. Apples, chocolate products, grapes, tea, legumes and wine could be identified as major food sources. As in Europe, differences in the habitual intake of proanthocyanidins among adult subgroups appeared to be associated with sociodemographic factors [30].

Whereas the isoflavonoid intake from soy products by Asian populations is relatively well documented [31–34], data on the consumption of monomeric and oligomeric flavanols in Asian countries are lacking. A few studies report daily amounts of total flavonoids in specific Asian countries without specifying the intake of oligomeric flavan-3-ols. For example, in a population of almost 1400 adult Chinese total flavonoid intake was assessed as 166 mg/d [35]. A case-control study in Korea middle-aged men revealed that on average 105 mg total flavonoids were daily consumed of which 20–23 mg were flavan-3-ols [36]. However, these data do not provide an estimate of the ingested proanthocyanidin quantities, because the reported flavan-3-ols only comprised monomers. Similarly, a cross-sectional cohort study including 569 middle-aged Japanese women estimated flavan-3-ol monomer consumption by means of 24 h food records as 386 mg flavan-3-ols (i.e. (-)-epicatechin, catechin and epicatechingallate) [37]. Ninety-eight percent of these flavanols were ingested by tea.

Australian adults were found to daily consume on average 454 mg total flavonoids of which 92% are flavan-3-ols (without any subclass specification) predominantly derived from tea consumption [38]. A prospective clinical study in 948 Australian women above 75 years assessed by means of food frequency questionnaires an average total proanthocyanidin (sum of dimers, trimers, 4–6 mers, 7–10 mers, polymers) intake of 215 mg/d (range: 18–1728 mg/d) which was mainly associated with the consumption of fruits, chocolate and alcoholic beverages [39].

Proanthocyanidin intake data for Middle and South America can hardly be found. Arabbi et al. determined the flavonoid content of commonly consumed Brazilian fruits and vegetables [40]. They also gave an overview on 4 epidemiological studies that indicate that the total habitual flavonoid intake in Brazilians ranges between 60 and 106 mg/d. They concluded from these studies that dietary flavonoid sources in Brazil are not very diverse since more than 70% of flavonoids are derived from oranges, 12% from lettuce and about 3% from tomatoes [40]. A recent survey estimates total average flavonoid intakes of 139 mg/d [41]. Despite a lack of data on quantities of subclasses, the identified major flavonoid food sources included legumes, fruits and beverages which are known to be rich in proanthocyanidins [24, 25, 42]. However, the total habitual intake of fruits and vegetables in Brazil appears to be much lower than in other countries world-wide. Less than 10% of the Brazilian population daily consumes 400 g fruits and vegetables as recommended by the WHO [40, 43]. Data on dietary flavonoid intake in African countries are not available in literature. In view of the discrepancies in chemical nomenclature, the size and anthropometric composition of the cohorts, the dietary assessment of food intake, the estimation of flavonoid and proanthocyanidin content of national food products by the use of different flavonoid databases, it is obvious that the reviewed data provide only rough estimates of the actual dietary intake of monomeric and oligomeric proanthocyanidins around the globe. However, it can be concluded with confidence that monomeric and oligomeric flavan-3-ols are present in human diets world-wide. Regular consumption of pome and stone fruits, berries, legumes, tea, wine and dark chocolate products leads to an intake of several hundreds of mg per day.

*Due to the methodological limitation being associated with the quantitative assessment of the daily consumption of specific dietary constituents within populations, it is likely that individual’s intake may be above or below the population’s average. Since the daily dietary intake of monomeric and oligomeric flavanols fluctuates in and between individuals the attribution of health benefits to daily intakes remains problematic. Therefore, a nutraceutical with a well-defined monomeric and oligomeric composition, such as Masquelier’s® Original OPCs, constitutes a relevant alternative since its recommended use will provide the health benefits that were established in preparation-specific research.*

### Chemical composition of Masquelier’s grape seed extract

In order to warrant safety and efficacy of a plant extract, i.e. pharmaceutical quality and physiological consistency, the same criteria as for synthetic substances apply, i.e. identity, purity and content of the material must be established batch-to-batch [44]. Quality assurance of plant extracts is challenging since the raw materials from which they are obtained are prone to a number of variables that cannot always be controlled. The spectrum of constituents of natural materials relies on environmental factors under which the plant is grown (e.g. soil composition, climate, pest) as well as processing procedures (e.g. harvesting, transportation, drying, storage). Masquelier’s® Original OPCs exemplify how pharmaceutical-grade quality of a plant extract can be achieved. Table 1 provides an overview on the properties and methods used to assess the identity, composition and purity of the extract. The preparation’s identity is established by organoleptic and spectrophotometric characteristics, e.g. the polyphenols’ absorbance maxima of λ = 220–280 nm. The purity of the preparation is determined by moisture, ash, physical constants, solvent residues, microbiological contaminations, foreign materials (e.g. heavy metals, pesticide residues, aflatoxins) and adulterations (Table 1).

**Table 1:** Properties to control identity and purity of Masquelier’s grape seed extract. The data were provided by the extract’s manufacturer

Property	Standard	Method
Organoleptic characteristics		
Aspect	Fine powder	
Color	Light brown/pinkish	
Odor	Characteristic woody	
Taste	Astringent, bitter	
Identification		In house
UV spectrum (approx. 40 mg/L in ethanol 96%)	λ max = 280 ± 5 nm	
	λ min = 256 ± 5 nm	
Bate-Smith reaction (approx. 100 mg/L)	λ max = 540–550 nm	
Loss on drying	≤10%	Ph. Eur. 01/2005: 20817
Sulphated ashes	≤0.5%	Ph. Eur. 01/2005: 20414
Solubility	conform	Ph. Eur. 6.7: 51100
1% (w/v) in water	insoluble < 5%	
2% (w/v) in methanol	Soluble	
1% (w/v) in isopropanol	Soluble	
0.01% (w/v) in chloroform	Insoluble	
pH (4% (w/v) in water)	2.5–4.5	In house
Residual solvents	conform	Ph. Eur. 6.7: 20424
acetone	≤10 ppm	
chloroform	≤10 ppm	
ethanol	≤10 ppm	
ethyl acetate	≤10 ppm	
n-butanol	≤10 ppm	
Microbiology	conform	Ph. Eur. 6.7: 50104
Bacteria	<10000 cfu/g	
*Enterobacteria*	<100 cfu/g	
*Escherichia coli*	0 cfu/g	
*Staphylococcus aureus*	0 cfu/g	
*Salmonella*	0 cfu/10 g	
*Pseudomonas aeruginosa*	0 cfu/g	
Yeasts and moulds	<100 cfu/g	
Mycotoxins	Comply with Reg. EC 1881/2006	In house
Pesticide/phytosanitary products ^a^	≤0.05 ppm	Ph. Eur. 6.7: 20813
Heavy metals (Fe, Cd, Hg, Pb, As)	<1 ppm	Ph. Eur. 6.7: 20408
Polychlorinated biphenyls and dioxins	Comply with Reg. EC 1881/2006	In house

^a^ The EU has established Maximum Residue Limits (MRL) of pesticides for “*certain products of plant origin*” (Directive 90/642/EC incl. amendments and corrections). The investigated phytosanitary products include aldrin; 4,4’-dichlorodiphenyldichloroethane; 4,4’- dichlorodiphenyldichloroethylene; 2,4’- dichlorodiphenyltrichloroethane; 4,4’- dichlorodiphenyltrichloroethane; dieldrin; α-endosulfan; β-endosulfan; endrin; α-hexachlorocyclohexane; β-hexachlorocyclohexane; γ-hexachlorocyclohexane; heptachlor; trans-heptachlor epoxide; hexachlorobenzene; methoxychlor

The extract’s specification of the content requires the qualitative and quantitative analysis of the phytochemicals. Various colorimetric though unspecific assays have a long tradition to determine the presence of polyphenols in general. The Folin-Denis and the Folin-Ciocalteau reagents, respectively are largely used to quantify total polyphenolic content of plant materials [45]. Both reagents rely on the oxidation of phenolic compounds in alkaline solution and subsequent reaction to blue colored phosphotungstic-phosphomolybdenum complexes which absorb at a wavelength of around λ = 760 nm. By means of the reference polyphenol gallic acid total polyphenolics are quantified as gallic acid equivalents (GAE) per kg or liter extract. As shown in Table 2 the total polyphenolic content of Masquelier’s grape seed extract amounts to 90–95% according to spectrophotometry and the Folin-Ciocalteau method. Drawbacks of this method are that not all plant phenolics are detected with the same sensitivity and that with regard to mono- and/or oligomeric flavanols, it is useless since it is unspecific and cannot provide any information in terms of the various flavanolic fractions in extracts. Moreover, sugars, aromatic amines and ascorbic acid can interfere with the color reaction [46].

**Table 2:** Analysis of the flavanol content of Masquelier’s grape seed extract. The data were provided by the extract’s manufacturer

Compound	Analytical method	Content
Total polyphenols	Spectrophotometric (GAE), *n* = 3	90–95%
	Folin-Ciocalteau reagent (GAE), *n* = 3	
Flavan-3-ols	Vanillin-H_2_SO_4_ (Sun et al., [50]), *n* = 9	60–70%
	HPLC, *n* = 14	
Monomeric flavanols	HPLC (every batch)	25–30%
Oligomeric flavanols (2–5 units)	HPLC (every batch)	35–40%
Polymeric flavanols (>6 units)	HPLC (every batch)	1–5%

*GAE* gallic acid equivalence, *HPLC* high performance liquid chromatography

**Fig. 4:**
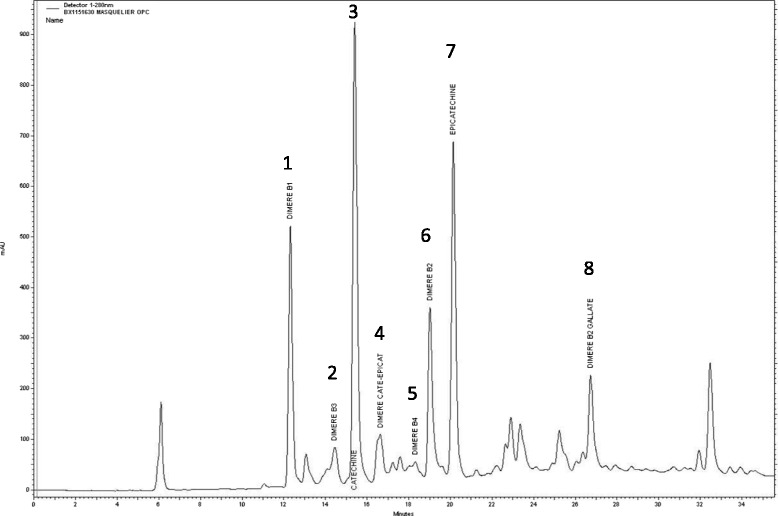
Representative chromatogram of Masquelier’s grape seed extract measured by high performance liquid chromatography (HPLC) using a C18 base-deactivated reversed-phase column (e.g. ProntoSIL 120-5-C18H, Bischoff Chromatography, Leonberg, Germany) with a gradient elution system of water/trifluoroacetic acid (TFA) 0.005% (v/v) and acetonitrile 65% (v/v)/TFA 0.005% (v/v) at a flow rate of 0.7 ml/min, at ambient column temperature and with UV detection at a wavelength of λ = 280 nm. The peaks in the chromatogram have been identified as the following individual monomeric and oligomeric flavan-3-ols: 1 = procyanidin B1, 2 = procyanidin B3, 3 = (+)-catechin, 4 = dimeric procyanidin consisting of (+)-catechin-(-)-epicatechin, 5 = procyanidin B4, 6 = procyanidin B2, 7 = (-)-epicatechin, 8 = prodcyanidin B2 gallate. The data were provided by the extract’s manufacturer

**Fig. 5:**
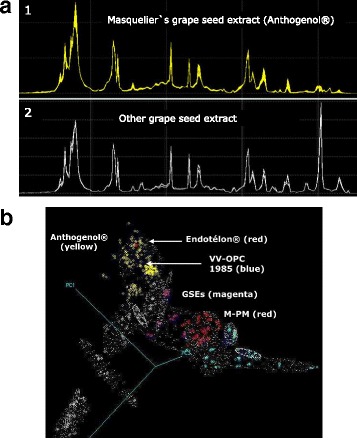
**a** Typical examples of an unassigned fingerprint proton nuclear magnetic resonance (^1^H-NMR) spectra of Masquelier’s grape seed extract (1) and another grape seed extract (2). **b** Three-dimensional plot of the principle component analysis (PCA) using unassigned fingerprint proton nuclear magnetic resonance (^1^H-NMR) spectra to compare the phytochemical content of different plant extracts e.g. from distinct manufacturers or from different batches. The small white dotted lines indicate the 95% confidence interval per cluster. The cluster of Masquelier’s grape seed extract (Anthogenol®, *yellow*) clearly distinguish from the cluster of French pine bark extract (M-PM, *red*) and other grape seed extracts (GSEs, magenta). Samples of the Endotélon extract (*red*) and samples of Masquelier’s extract from 1985 (VV-OPC 1985, *yellow*) are located within the cluster of Masquelier’s grape seed extract proofing the qualitative and quantitative equality of these extracts. The green-blue lines represent the 3 strongest principal components (PC), i.e. the variables derived from the ^1^H-NMR spectra which define the biggest differences between all the samples. The farther 2 samples are apart from each other in respect of one particular axis, the more different they are in this particular aspect. The data were provided by the extract’s manufacturer

The commonly used colorimetric method to determine the presence of proanthocyanidins is n-butanol/HCl hydrolysis to red colored anthocyanidins which have an absorbance maximum at around λ = 550 nm [47, 48]. Due to numerous technical limitations it was recommended to use this assay to detect the presence of oligomeric flavanols rather than to quantify them [49]. Another limitation is that the Bate-Smith reaction does not differentiate between specific fractions of proanthocyanidins. The coloration is produced by all proanthocyanidins, oligomers and polymers, i.e. irrespective of their level of polymerisation. For the purpose of quantifying total proanthocyanidins in a preparation, the vanillin assay might be a slightly better colorimetric alternative. In the presence of sulfuric acid vanillin reacts with condensed flavan-3-ols (oligomers and polymers) to a red colored complex that can be measured at a wavelength of λ = 500 nm. Usually, catechin is used as a reference substance although for the quantification of polymerized flavanols internal standards would be more advantageous to account for different reaction rates of monomers and oligomers [50]. Various factors influencing the outcomes of the vanillin assay were identified [49], and led to a meticulous reexamination of the assay [50]. By means of this vanillin method the total proanthocyanidins content of Masquelier’s Original OPCs amount to 60–70% (Table 2). Nonetheless, the major disadvantage of all these spectrophotometric assays is that they solely provide an estimation of the total polyphenolic and/or proanthocyanidin content of extracts. Individual fractions cannot be qualified or quantified by these methods. For this purpose, high performance liquid chromatography (HPLC) is the preferred technique. Several excellent reviews have been published over the last years that provide an overview on HPLC methods for the quantification of polyphenols as wells as a critical appraisal of factors that are essential to take into account for reliable measurements [46, 51–53]. In 1990, Masquelier and his team developed an HPLC method to analyze the monomeric and oligomeric flavanol content in grape seed and pine bark extracts [54]. To isolate and identify the individual monomeric and oligomeric flavanols the crude plant extract was first purified by solid phase extraction (SPE) on Sephadex G25 and LH20 columns and by semi-preparative HPLC. Fifteen flavanol monomers and oligomers could be identified by chemical (acidic degradation in the presence of α-toluenethiol) and spectroscopic techniques like nuclear magnetic resonance (NMR), ultraviolet-visible (UV/VIS) and infrared (IR) [55]. Next to the monomers (+)-catechin, (-)-epicatechin and (-)-epicatechin gallate, 6 dimers of the B-series were found, i.e. proanthocyanidin B1, B2, B3, B4, B5 and B7 and 1 dimer of the A-series, i.e. proanthocyanidin A2. Moreover 4 trimers have been identified, i.e. (+)-catechin - (+)-catechin - (+)-catechin, (-)-epicatechin - (-)-epicatechin - (-)-epicatechin, (-)-epicatechin - (-)-epicatechin - (+) catechin and (-)-epicatechin - (-)-epicatechin - (-)-epicatechin-gallate (EEEg) as well as a tetramer consisting of (-)-epicatechin - (-)-epicatechin - (-)-epicatechin - (-)-epicatechin [54]. Later, the preparation’s manufacturer (Berkem SA; Gardonne; France) in collaboration with the University of Bordeaux repeated and modified Masquelier’s analyses by using a C18 base-deactivated reversed-phase column (e.g. ProntoSIL 120-5-C18H, Bischoff Chromatography, Leonberg, Germany) with a gradient elution system of water/trifluoroacetic acid (TFA) 0.005% (v/v) and acetonitrile 65% (v/v)/TFA 0.005% (v/v) at a flow rate of 0.7 ml/min, at ambient column temperature and with UV detection at a wavelength of λ = 280 nm. In the chromatogram obtained mainly the monomeric and dimeric compounds are identified (Fig. 4), although centrifugal partition chromatography (CPC), preparative HPLC, NMR and mass spectrometry allowed the identification of 14 monomeric, dimeric and trimeric flavan-3-ols in addition to gallic acid. This HPLC analysis can be used to routinely monitor the composition of the grape seed extract next to ^1^H-NMR spectroscopy followed by principle component analysis (PCA) as described below. Batch-to-batch analyses revealed that approximately a fourth of the grape seed extract’s flavanols is made up by monomers, a second fourth are dimers and half of the extract consists of trimers, tetra- and pentamers (Table 3). Normal phase (NP)-HPLC demonstrated the absence of higher polymerized flavanols [56]. The qualitative and quantitative analysis of the grape seed extract is an important prerequisite to control and achieve a constant, standardized phytochemical composition. The HPLC data revealed that the Masquelier’s extract is a complex mixture of individual flavanols of various polymerization degrees (*n* = 2–5). Monitoring batch-to-batch variations of the preparation based on only one flavanol would be inadequate because changes in one specific flavanol might not be representative for all the different individual flavanolic fractions. Since the physiologically active principle of a plant extract is the entire mixture of individual phytochemicals and not a single compound, profiling the whole spectrum of phytochemicals in the grape seed extract is necessary to guarantee a stable composition and ultimately consistent bioactivity. NMR spectroscopy is a versatile analytical technique to screen complex composed samples with minimal and non-destructive sample preparation. Standard ^1^H- or ^13^C-NMR spectra provide a wealth of information on both, chemical structures of individual compounds and chemical profiles of compound mixtures. That makes it increasingly popular for elucidation of novel plant-derived compounds as well as for characterization of plant extracts and food products [52]. All molecules containing atoms with a non-zero magnetic moment (e.g. ^1^H, ^13^C, ^14^N, ^15^N, ^19^F, ^31^P) give an unique NMR signal which originates from the structural location of these atoms in the molecule. NMR analysis of an extract with a complex mixture of plant metabolites provides a unique spectrum of signals, i.e. a fingerprint, depending on its molecular composition (Fig. 5a). Multivariate analysis of such unassigned fingerprint NMR-spectra is a convenient and accurate tool to compare the phytochemical content of different plant extracts e.g. from distinct manufacturers or from different batches [57]. Therefore, ^1^H-NMR spectroscopy followed by PCA [58] is used to control batch-to-batch consistency of Masquelier’s grape seed extract. The yellow dots in the three-dimensional plot (Fig. 5b) represent the 95% confidence intervals of each batch analysis and form a clearly separated cloud indicating high similarity of the phytochemical NMR-profile of the individual batches. This cluster of Masquelier`s grape seed extract batches (Anthogenol®, yellow) clearly distinguishes from the cluster of French pine bark extracts (M-PM, red) and other grape seed extracts (GSEs, magenta). Samples of the Endotélon extract (red) and samples of Masquelier’s extract from 1985 (VV-OPC 1985, yellow) are located within the “Anthogenol” cluster proving the qualitative and quantitative equality of these extracts and its consistency throughout the years. The application of NMR fingerprinting enables to continuously authenticate the extract and allows for the production of a genuinely standardized product. Eventually, this is essential for safeguarding the preparation’s physiological consistency.

**Table 3:** Mean quantities ± standard deviation (SD) of monomeric and oligomeric flavan-3-ols of 10 batches of Masquelier’s grape seed extract measured by high performance liquid chromatography (HPLC, see also Fig. 4). The data were provided by the extract’s manufacturer

Compound	Mean quantity ± SD (% (w/w))
*Total monomers*	*25.6* ± *2.2*
(+)-catechin	10.9 ± 1.6
(-)-epicatechin	12.2 ± 1.6
(-)-epicatechin-3-O-gallate	2.5 ± 1.6
*Total dimers*	*27.5* ± *1.6*
Procyanidin B1	7.7 ± 1.6
(-)-epicatechin-(4 β → 8)-(+)-catechin	
Procyanidin B2	8.3 ± 1.6
(-)-epicatechin-(4β → 8)-(-)-epicatechin	
Procyanidin B3	2.8 ± 1.6
(+)-catechin-(4α → 8)-(+)-catechin	
Procyanidin B4	1.6 ± 1.6
(+)-catechin-(4α → 8)-(-)-epicatechin	
Procyanidin B2-gallate	7.1 ± 1.6
*Total tri*-, *tetra*- *and pentameric proanthocyanidins*	*46.9* ± *1.6*

*Lacking standardization and characterization of the phytochemical composition of herbal extracts makes studying the* in vivo *biotransformation of the plant*-*derived constituents cumbersome. With a meticulously defined product*, *such as Masquelier*’*s flavanol blend*, *it becomes feasible to study the bioavailability and metabolism*.

### Bioavailability and metabolism of Masqulier’s grape seed extract

Knowledge on the bioavailability, biotransformation and kinetics of grape-seed derived flavan-3-ols is important for the elucidation of their mechanism of action. In addition, these data strengthen the cause-effect relationship between the (recommended) dietary intake and the observed health effect of these compounds. In order to shed more light on the fate of orally ingested monomeric and oligomeric flavan-3-ols, animal experiments were conducted in the late 70ies of the last century. Radiolabeled monomeric and oligomeric flavan-3-ols (0.6 μCi (0.2 × 10^3 Bq)/mg) could be obtained from the seeds of grapes (*Vitis vinifera* L. of the Cabernet and Merlot variety) grown during grape formation in a ^14^CO_2_ atmosphere for 40 days. After oral administration of a single dose of 2 μCi (approximately 3.3 mg grape seed extract) radioactivity in blood of mice (*n* = 5) was measured at 9 consecutive time points between 10 min and 7 h [59, 60]. Following a rapid increase in the blood’s radioactivity, maximal plasma levels were reached 45 min. after ingestion. The plasma half-life was estimated to 5 h. The distribution of the flavanols in the mice was determined in various types of tissue collected at 1, 3, 6 and 24 h after intake of a single oral dose (5 mg) [59, 60]. During the first 6 h most of the radioactivity was located in the gastro-intestinal tract including gall-bladder and bladder. Lower levels of radioactivity could be detected in the liver, kidneys, skin, cartilage tissue, arteries, cardiac muscle and blood. After 24 h the gastro-intestinal tract was free of any radioactivity. In contrast, radioactivity was markedly elevated in the gastro-intestinal vasculature, arteries, skin, and cartilage tissue. Decreasing levels of radioactivity were seen in liver, spleen, venous tunica, periost, kidneys, lungs, bones and blood. Repeated daily dosages over 5 consecutive days did not seem to lead to an accumulation of the flavanols in mice [59, 60]. In rats (*n* = 5) bile was found to be essentially involved in the elimination of monomeric and oligomeric flavanols [59, 60]. The use of semi-synthetically produced ^3^H-labeled flavan-3,4-diol monomer confirmed the tissue distribution of the ^14^C-labeled grape seed flavanols. Since ^3^H-radioactivity remained measurable in mice tissue sections, especially in collagen-rich types of tissue, it was suggested that tissue binding of flavanols neither involves the hydroxyl-group in the C4-position of the flavan-moeity (C-ring) nor *in situ* polymerization of flavanol-monomers via the C4-position [59, 60]. Although these early experimental data indicate that grape-seed-derived monomeric and oligomeric flavanols become bioavailable upon oral ingestion, it remained unknown in which form, i.e. as parent compounds or metabolites, they are distributed in the body. Moreover, it remains to be elucidated whether bioavailability and biotransformation routes differ between rodents and humans as reported for other types of flavonoids [61].

In a preliminary human pharmacokinetic study with ^14^C labelled grape seed mono- and oligomeric flavanols (Endotélon®), 6 healthy volunteers received a single oral dose of 150 mg in the morning under fasting conditions [62]. Plasma radioactivity was in general low and found to be in the range of ng equivalents of the administered dose. Despite considerable inter-individual differences maximal radioactivity plasma levels were observed at approximately 14 h after flavanol administration with a slow decrease over the subsequent 144 h. During 96 h after intake between 11 and 27% (mean ± SD = 22.09 ± 5.99%) of the oral dose was eliminated via urine and between 22 and 67% (mean ± SD = 45.61 ± 14.92%) via feces. On average approximately 6% of the flavanols was measured in breath as ^14^CO_2_ with the majority exhaled during the first 24 h [62].

One major drawback of those studies is that they do not provide any information on the origin of the ^14^C labelled radioactivity in tissue, i.e. whether it derives from the ingested parent compounds or from metabolites. Thereby it remains unclear in which form flavanols reach target tissue and may be responsible for putative health effects. The development of highly sensitive analytical techniques such as HPLC coupled to mass spectrometric (MS) detection has considerably increased insights in the metabolic fate of monomeric and oligomeric flavanols. Today it is possible to quantify flavanols in the fmolar range in various types of matrices and at the same time to elucidate their molecular structure. This has considerably advanced the knowledge on the absorption, distribution, metabolism and excretion of flavanols. Animal studies reported flavanolic monomers, dimers and trimers in plasma, urine and feces of rats orally administered with grape seed extracts [63–66]. In these in vivo studies the major biotransformation routes comprised methylation, glucuronidation and sulphation of both, monomeric and oligomeric flavanols [64–66]. Data on the bioavailability and metabolism of grape seed derived flavanols in humans are scarce. A recent perfusion study revealed that the absorption of a single 50 mg dose of the flavanolic monomer (-)-epicatechin varies between healthy persons ranging between 31 and 90% [67]. Glucuronidation, sulphation and methylation produce the major epicatechin metabolites that can be found in plasma approximately 2 h after oral ingestion [68] and which are excreted in bile and in urine [67]. Plasma concentrations of untransformed epicatechin have been reported to be around 1% of the epicatechin sulfate metabolites and amount to approximately 4 nM 1 h after ingestion of a cocoa dairy-based drink providing 1.8 mg epicatechin per kg body weight [68]. In contrast to monomers human data on the fate of oligomers are less consistent. Dimeric B-type proanthocyanidins have been detected in nmolar quantities in the circulation after a single dose of 323 mg cocoa-derived monomers and 256 mg dimers [69] and 2 g of a grape-seed extract [70], respectively. However, higher condensation products are assumed not to be absorbed due to their molecular weight, although in vitro and in vivo studies showed the absorption of the proanthocyanidins trimer C2 [64, 66, 71]. Trimeric and larger condensation products are generally suggested to undergo colonic degradation to phenolic acids [72] and valerolactones [73, 74], rather than to serve as monomer precursors [68, 75]. Therefore, based on the current data it needs to be presumed that the intake of grape seed extract containing both, monomeric and oligomeric flavan-3-ols, will lead to monomeric phase II conjugates and colonic biotransformation products in the human circulation. It remains to be elucidated in how far these metabolites contribute to the observed health effects.

*Over the past decades*, *the state of the art of analytical*-*chemical techniques increased considerably. These technical advances revealed that the historical data using radioactive*-*labelled grape seed flavanols lacks specificity. Thereby*, *the precise metabolic fate and kinetic profile of Masquelier*’*s grape seed extract is still incomplete. Whether flavanol*-*tissue binding takes place as concluded by Laparra* et al. [59, 60], *remains questionable. Still*, *the fact that proanthocyanidins bind to collagen*-*rich tissues has been well established. Polymers of proanthocyanidins are called tannins because of their* “*tanning*” *properties. Tannins strongly bind to collagen*-*rich tissue* (*leather*). *This millenia*-*old knowledge forms an indication that part of the oligomers*’ *protective mode of action may stem from their affinity to collagen. In this regard*, *separate from the kinetic profiling of flavanolic plant extracts*, *their physiological mode of action is key in developing the product as a nutraceutical*.

### Mechanisms of action of Masquelier’s grape seed extract

#### Effects on collagen and elastin fibers in vitro and in vivo

The time-journey of Masquelier’s monomeric and oligomeric flavan-3-ols extract started with the observation of their effect in vascular tissue. With other grape seed extracts many other, often similar studies have been done. It is, however, known that grape seed extract may vary widely in the way they are composed (*vide supra*) as nicely illustrated by Nakamura et al. [76]. It is therefore important to use a well-characterized grape seed extract with a stable composition. Since composition affects biological activity, in the description of the mechanisms of action we limit this review to the available data on Masquelier’s extract. The microcirculatory effect of Masquelier’s monomeric and oligomeric flavanol extract was correlated to the original findings that the preparation protects collagen. The stability of collagen depends on the cross-linking of the peptic chain in the molecule. Denaturation of the molecule, for example by increased temperature or oxidative stress, leads to shortening of the collagen fibers. In vitro, Masquelier’s® Original OPCs have been shown to protect against the thermal contraction of the collagen [77]. Of all bioflavonoids only the proanthocyanidins (oligomeric flavanols) seem to exert this protecting effect which might be due to the variation in their cross-linking effect. Oligomeric flavanols show strong collagen protection and isotopic labelling of monomeric and oligomeric flavan-3-ols can be interpreted as a confirmation of the oligomers’ affinity for the vascular wall (*vide supra*) [60]. This was also confirmed in 1982 by A. Pfister et al. who studied the localization of monomeric and oligomeric flavanols in cell membranes of endothelial cells and pneumocytes [78].

Similarly, the risk of vascular damage can be reduced by Masquelier’s grape seed extract through the protection of elastin from degradation by elastase in vitro and in vivo. In vitro, the solubilization of elastin obtained from calf articular ligament by either elastase from porcine pancreas or human leukocyte was inhibited by incubating the elastin with the extract. Also in vivo, after intradermal injections of the flavanols in the skin of rabbits, it was shown that this pretreatment protected the elastin [79]. The inhibition of the hydrolysis of elastin by elastase and of collagen by collagenase has been confirmed in other in vitro models [80]. There are even indications that the synthesis of collagen and elastonectin fibers is promoted by the extract in vitro [80] and in vivo [78].

This antiproteolytic activity offered by the oligomeric flavan-3-ols present in the preparation has been classified as ‘substrate protection’, because the compounds do not primarily inhibit the proteinases that degrade the matrix-components but rather bind to the matrix macromolecules thus preventing degradation by various triggers such as temperature, oxidative stress, inflammation, proteinases among others [81].

In Wistar rats, collagenase-induced vascular capillary hyperpermeability was inhibited by the grape seed derived monomeric and oligomeric flavanols [80]. The flavanols’ strengthening effect on collagen- and elastin-rich tissues like blood vessels and the venous system has also been demonstrated in various human intervention studies as reviewed in 2003 [82].

#### Antioxidant effects in vitro and in vivo

With the discovery that reactive oxygen species play a pivotal role in the aging physiology a lot of attention was given to this research area [83]. Also vascular function is strongly influenced by oxidative stress [84]. The imaginative studies in the early fifties of the previous century already demonstrated that the oxidation of the antioxidant vitamin C could be prevented by Masquelier’s extract [85]. The established action of the flavanols on vascular function directed the research to the field of prevention and attenuation of oxidative stress and ever since the antioxidant action of monomeric and oligomeric flavan-3-ols has indeed been widely studied. In 1987, the US Patent Office granted Jack Masquelier a patent for the use of proanthocyanidins as antioxidants in various fields of human physiology [86].

Polyphenolic structures are known to be good antioxidants [87]. Monomeric and oligomeric flavanols have been shown to have radical scavenging activity, e.g. scavenging of the 2,2-diphenyl-1-picrylhydrazyl (DPPH) radical [88] and the superoxide anion radical [89, 90]. Moreover, monomeric and oligomeric flavan-3-ols protect against hydrogen peroxide [91] and organic peroxide (*i.e*. cumene hydroperoxide)-induced damage of fetal bovine vascular endothelial cells [92]. Iron (Fe^2+^- adenosine diphosphate (ADP)) in combination with ascorbic acid leads to lipid peroxidation in rat liver microsomes, which is inhibited by monomeric and oligomeric flavan-3-ols [89, 93].

Monomeric and oligomeric flavan-3-ols are clearly and not unexpectedly (based on the molecular structure of the constituents) able to scavenge reactive oxygen and nitrogen species and inhibit lipid peroxidation. Due to the low concentrations that are found in the human circulation after flavanol supplementations (*vide supra*), the relevance of direct antioxidant effects of these compounds under physiological conditions is increasingly debated. However, we recently demonstrated that also epicatechin phase II metabolites attenuate intracellular oxidative stress in primary human umbilical vein endothelial cells (HUVECs) in concentrations as low as 0.5 μM [94].

With the progressing insights into redox regulation of cell signaling during the last decades, studies aimed at unraveling the effects of monomeric and oligomeric flavanols on redox-sensitive transcription factors like nuclear factor (erythroid-derived 2)-like 2 (Nrf2). Nrf2 is critical in the transcriptional regulation of genes coding enzymes that facilitate cellular protection against oxidative stress. In hepatocytes it was recently shown that epicatechin transiently activates, among other redox-sensitive transcription factors, Nrf2 [95]. Similarly, procyanidin B2 activated redox-sensitive signaling cascades involved in the activation of Nrf2, induced the translocation of Nrf2 from the cytoplasm into the nucleus and activated the transcription of the detoxifying enzyme glutathione S-transferase pi 1 (GSTP1) in in epithelial colon cells [96]. Interestingly, Nrf2 activation and modulation of Nrf2-mediate gene transcription was reported in various in vitro and in vivo models by monomeric and oligomeric flavanol-rich extracts of different plants sources [97–99] including grape seeds [100, 101].

Nowadays, it is well established that diminishing oxidative stress also reduces the inflammatory response. A vicious interplay between oxidative stress and inflammation is apparent. In fact, inflammation is probably fundamental in many pathologies and maintaining health is achieved by keeping the inflammation low.

#### Anti-inflammatory effects in vitro and in vivo

Monomeric and oligomeric flavan-3-ols inhibit the inflammatory response also in a more direct way. Leukocyte-induced luminescence stimulated with either zymosan or the phorbol ester phorbol12-myristate 13-acetate (PMA) was dose-dependently inhibited by the monomeric and oligomeric flavanols preparation [88]. Also in animal experiments the anti-inflammatory effect of Masquelier’s extract has been demonstrated. Blaszò et al. reported that in a carrageenan-induced rat paw oedema model the intraperitoneally administered preparation in dosages of 10, 20, 40 mg/kg displayed an anti-inflammatory action [102]. Oral administration of rats with 500 mg/kg Masquelier’s Original OPCs® also gave protection against the carrageenan paw oedema effect [88].

More than 20 years later, the anti-inflammatory effects of the grape-seed derived monomeric and oligomeric flavanols were further explored on a cellular and subcellular level. The transcription factor nuclear factor kappa-light-chain enhancer of activated B cells (NF-κB) is the key regulator of the transcription and synthesis of inflammatory mediators in immune cells [103]. In human monocytes (U937 cells) which were stably transfected with a luciferase-reporter gene construct containing NF-κB binding sites and which were differentiated into macrophage-like cells the inhibition of NF-κB-mediated gene expression by the grape-seed derived monomeric and oligomeric flavanols was demonstrated [104]. That this subcellular effect has consequences for the function of human monocytes was proven in an assay that allows studying the chemotaxis and adhesion of monocytes to vascular endothelial cells [104]. Preincubation of the monocytes with the grape-seed derived monomeric and oligomeric flavanols for 24 h reduced the number of adhering cells at the endothelial cells by approximately one third [104]. These data shed light on a molecular mechanism of the grape-seed derived flavanolic preparation that may help to explain their anti-inflammatory activity observed in humans (*vide infra*).

Moreover, (-)-epicatechin was recently suggested to enhance the anti-inflammatory effects of the endogenous glucocorticoid cortisol in human macrophage-like cells under oxidative stress [105]. Next to the parent compound also epicatechin phase II metabolites exhibit capacity to preserve the anti-inflammatory effects of cortisol in this model [106]. This mechanism is an eminent example of the intertwined link between oxidative stress and the development of inflammation by a compromised endogenous cortisol response. In how far Masquelier’s extract exert similar protection of the cortisol response must be demonstrated.

*As illustrated here*, *Masquelier*’*s flavanol blend show a multitude of biological effects. This is distinctive from most pharmaceuticals that are generally selected for their strong effect on a well*-*defined single molecular target. The efficacy of drugs in humans is determined in clinical trials by a statistical change in a primary outcome parameter indicative for an improvement of a disease. In contrast*, *to capture the multiple* - *pleiotropic* - *effects of botanical extracts a broad panel of biomarkers is required. Moreover*, *effects on human health are characterized by the ability to withstand a stressor in the physiological system and maintain or restore homeostasis* [107]. *The relevance of results from human intervention studies should therefore be judged from this perspective, i.e. whether the observed effects are the result of integrated physiological phenomena*.

### Effects of Masquelier’s grape seed extract on human health

Initially, several clinical studies on the protective effect of Endotélon® (the French herbal remedy consisting of Masquelier’s grape seed extract) on capillary fragility were performed. Dartenuc et al. reported in an open-label study using 100 – 150 mg/d of Endotélon® for 30 – 45 days a reduction in capillary fragility in 39 out of 46 subjects assessed by a cupping glass method [108]. To confirm these findings, the researchers performed a second study which was placebo-controlled. The daily intake of Masquelier’s grape seed preparation for 15 days improved capillary fragility in 10 of 21 volunteers, whereas in the placebo-group a similar effect was only seen in 3 out of 12 subjects [108]. The capillary resistance defined as the property of capillaries to counteract the forces of rupture was improved by the intake of 100–150 mg after 15–30 day of intervention [109]. The protection of capillary resistance by Endotélon® was also demonstrated in a double-blind placebo-controlled study in subjects with either spontaneous capillary fragility or acetylsalicylic acid-induced capillary fragility [109]. In a double-blind placebo-controlled trial the measurement of skin temperature as a measure of dermal circulation and rheographic parameters were used to show an improved venous tone in subjects who daily took 150 mg of Masquelier’s preparation (Endotélon®) for 45 days [110]. During 15–90 days Beylot C. and Bioulac P. administered patients with a variety of problems related to capillary fragility 150 mg Endotélon® per day and observed a decrease in fragility in 62 out of 78 subjects [111]. In patients with varying phlebologic disorders the influence of Endotélon® on edema, varicose veins, hypodermitis, capillaries and petechies was investigated by a placebo-controlled randomized clinical trial [112]. The daily intake of 150 mg Endotélon® over 4 weeks improved edema and varicose veins compared to the placebo treatment. In a homogenous study population of 92 patients with a mean age of 40 years and a venous pathology of in average 7 years obtained 300 mg Endotélon® for 4 weeks [113]. Clinical scoring of the amelioration was achieved in 75% of the patients (versus 41% in the placebo group, *P* < 0.01). Also, edema reduction was apparent.

The most recent clinical data of Masquelier’s grape seed extract (embodied in the food supplement Anthogenol®) were obtained in a double-blind randomized placebo-controlled clinical study in healthy volunteers [114]. This study aimed at capturing the diverse effects of the grape seed-derived monomeric and oligomeric flavanols observed in all the previous studies by a broad panel of outcome parameters reflecting vascular function as well as cellular and subcellular processes in the human vasculature associated with cardiovascular pathologies. For this purpose, 28 male healthy smokers were daily supplemented for 8 weeks with 200 mg Masquelier’s® Original OPCs. Neither macrovascular function (assessed as flow mediated dilation of the brachial artery) nor microvascular function (measured by laser-Doppler flowmetry) changed significantly during the 8 weeks intervention compared with the placebo-supplemented group. Individuals with elevated total cholesterol and low density lipoprotein (LDL) serum concentrations in the beginning of the trial revealed a 5% and 7% reduction (*P* < 0.05 vs. baseline), respectively after 8 weeks supplementation with Masquelier’s preparation. These effects could not be observed in a similar subgroup on placebo. Whereas platelet aggregability remained unaffected, a significant attenuation of the inflammatory responsiveness of leukocytes to *ex vivo* added bacterial endotoxin (compared to baseline and compared to the placebo intervention) was found in blood from volunteers after the 8 weeks supplementation with Masquelier’s® Original OPCs. While total antioxidant capacity of plasma and the lipid peroxidation marker 8-iso-prostglandin F2α did not change during the intervention, erythrocytes’ ratio of reduced to oxidized glutathione appeared to increase in the supplemented group compared to baseline. Integration of all the effects assessed by the meticulously selected parameter panel into a global vascular health index revealed a significant increase upon the 8 weeks supplementation with Masquelier’s grape seed extract compared to placebo. In order to elucidate the underlying molecular pathways which might be involved in the observed changes, RNA and DNA was isolated from leukocytes of a subset of the volunteers to determine genome wide changes in gene expression and DNA methylation [104]. The dietary intervention with Masquelier’s extract seemed to affect in particular the expression of genes associated with pathways involved in the regulation of inflammation and chemotaxis, adhesion and transendothelial migration of leukocytes. Experimental models substantiate these findings by demonstrating the extract-mediated inhibition of the inflammatory transcription factor NF-κB [104]. This mechanism may also contribute to the reduced adhesion of monocytes exposed to the grape seed-derived flavanols to vascular endothelial cells in vitro [104]. Despite clear effects of the daily supplementation with 200 mg Masquelier’s® Original OPCs over 8 weeks on the expression of genes associated with pathophysiological mechanisms in the cardiovascular system, the transcriptomic changes could not be related to alterations in the DNA methylation state due to high inter-individual variability in leukocytes DNA methylation [104].

This beneficial modulation of inflammatory, metabolic and redox pathways in the vasculature and the very recently reported effects of a grape seed extract on liver nicotine adenine dinucleotide (NAD^+^)-metabolism and expression of the protein deacetylase sirtuin 1 [115] may hold promise to extend the clinical investigations on this extract in the future also to hepatic, endocrine and cognitive health.

## Conclusion

The three requirements to substantiate the health benefit of a food product include 1) the characterization of the product, 2) the clear definition and understandable wording of the physiological benefit and 3) the plausible cause-effect relationship between the food product’s intake and the health effect.

Ad 1) In contrast to many commercially plant extracts Masquelier’s grape seed preparation is rigidly defined and standardized by HPLC and ^1^H-NMR/PCA fingerprinting. This combination of methods is optimal for monitoring the quality of plant extracts and is superior to the analysis of one or more individual “target” components of an extract.

Ad 2) The numerous human intervention studies that became available over time formed the basis for designing an intervention study that employed innovative bio-molecular techniques and reflects conceptual advances in nutritional science. This novel approach enabled to capture the pleiotropic effects of Masqulier’s grape seed extract on vascular health in humans [114]. This physiological benefit can be briefly worded as “maintenance of vascular homeostasis”.

Ad 3) Understanding the consistency, dose–response and biological plausibility of the association between the vascular health effects and Masquelier’s grape seed extract is based on the totality of the human intervention studies. These outcomes are substantiated by experimental studies that elucidate the molecular mechanisms of the herbal extract and some of its individual components. In this regard, the knowledge on the mode of action of flavanols in general parallels and supports the more refined and preparation-specific insights into the physiological processes described in the studies on the products that embody the specific monomeric and oligomeric flavanols extract developed by Jack Masquelier. In the quest for unravelling the molecular action of complex botanical preparations such as Masquelier’s® Original OPCs, studies are ongoing to enhance our existing knowledge and understanding in the intricately related fields of nutrition, physiology, health and disease. The preparation’s pleiotropic effects explain why and how it can be applied as an herbal remedy as well as a nutraceutical in the field of vascular health.

## Abbreviations

^1^H-NMR: Proton nuclear magnetic resonance
ADP: Adenosine diphosphate
ANR: Anthocyanidin reductase
ANS: Anthocyanidin synthase
BMI: Body mass index
CPC: Centrifugal partition chromatography
DAHP: 3-deoxy-D-arabinose-heptulosonic acid-7-phosphate
D-erythrose-P: D-erythrose-4-phosphate
DFR: Dihydroflavanol-4-reductase
DPPH: 2,2-diphenyl-1-picrylhydrazyl
F3’H: Flavanone-3’-hydroxylase
F3H: Flavanone-3-hydroxylase
GAE: Gallic acid equivalence
GSTP1: Glutathione S-transferase pi 1
HPLC: High performance liquid chromatography
HUVECs: Human umbilical vein endothelial cells
IR: Infrared
LAR: Leucoanthocyanidin reductase
LDL: Low density lipoprotein
MS: Mass spectrometry
NAD: Nicotine-adenine dinucleotide
NADPH: Reduced nicotine-adenine dinucleotide phosphate
NF-kB: Nuclear factor kappa-light-chain enhancer of activated B cells
Nrf2: Nuclear factor (erythroid-derived 2)-like 2
OPCs: Oligomeric proanthocyanidins
PC: Principle components
PCA: Principle component analysis
PEP: Phosphoenol pyruvate
PMA: Phorbol12-myristate 13-acetate
PPO: Polyphenol oxidase
SPE: Solid phase extraction
TFA: Trifluoroacetic acid
UV/VIS: Ultraviolet-visible
